# A Novel Strategy for TNF-Alpha Production by 2-APB Induced Downregulated SOCE and Upregulated HSP70 in *O*. *tsutsugamushi*-Infected Human Macrophages

**DOI:** 10.1371/journal.pone.0159299

**Published:** 2016-07-29

**Authors:** Ching-Ying Wu, Wen-Li Hsu, Chun-Hsiung Wang, Jui-Lin Liang, Ming-Hsien Tsai, Chia-Jung Yen, Hsiu-Wen Li, Siou-Jin Chiu, Chung-Hsing Chang, Yaw-Bin Huang, Ming-Wei Lin, Tohru Yoshioka

**Affiliations:** 1 Graduate Institute of Medicine, Kaohsiung Medical University, Kaohsiung, Taiwan; 2 Lipid Science and Aging Research Center, Kaohsiung Medical University, Kaohsiung, Taiwan; 3 Center for Lipid Biosciences, Kaohsiung Medical University Hospital, Kaohsiung Medical University, Kaohsiung, Taiwan; 4 School of Medicine, Kaohsiung Medical University, Kaohsiung, Taiwan; 5 Department of Dermatology, Kaohsiung Medical University, Kaohsiung, Taiwan; 6 School of Pharmacy, Kaohsiung Medical University, Kaohsiung, Taiwan; 7 Center for Stem Cell Research, Kaohsiung Medical University, Kaohsiung, Taiwan; 8 Department of Dermatology, Kaohsiung Municipal Ta-Tung Hospital, Kaohsiung, Taiwan; 9 The Institute of Basic Medical Sciences, College of Medicine, National Cheng Kung University, Tainan, Taiwan; 10 Institute of Chemistry, Academia Sinica, Taipei, Taiwan; 11 Institute of Physics, Academia Sinica, Taipei, Taiwan; 12 Chi Mei Medical Center, Liouying, Tainan, Taiwan; Waseda University, JAPAN

## Abstract

*Orientia* (*O*.) *tsutsugamushi*-induced scrub typhus is endemic across many regions of Asia and the Western Pacific, where an estimated 1 million cases occur each year; the majority of patients infected with *O*. *tsutsugamushi* end up with a cytokine storm from a severe inflammatory response. Previous reports have indicated that blocking tumor necrosis factor (TNF)-α reduced cell injury from a cytokine storm. Since TNF-α production is known to be associated with intracellular Ca^2+^ elevation, we examined the effect of store-operated Ca^2+^ entry (SOCE) inhibitors on TNF-α production in *O*. *tsutsugamushi*-infected macrophages. We found that 2-aminoethoxydiphenyl borate (2-APB), but not SKF96365, facilitates the suppression of Ca^2+^ mobilization via the interruption of Orai1 expression in *O*. *tsutsugamushi*-infected macrophages. Due to the decrease of Ca^2+^ elevation, the expression of TNF-α and its release from macrophages was repressed by 2-APB. In addition, a novel role of 2-APB was found in macrophages that causes the upregulation of heat shock protein 70 (HSP70) expression associated with ERK activation; upregulated TNF-α production in the case of knockdown HSP70 was inhibited with 2-APB treatment. Furthermore, elevated HSP70 formation unexpectedly did not help the cell survival of *O*. *tsutsugamushi*-infected macrophages. In conclusion, the parallelism between downregulated Ca^2+^ mobilization via SOCE and upregulated HSP70 after treatment with 2-APB against TNF-α production was found to efficiently attenuate an *O*. *tsutsugamushi*-induced severe inflammatory response.

## Introduction

The immune system defends the body from infectious pathogens in two major ways: innate and adaptive immunity. The innate immune system is the first line of host defense against invading organisms, with the adaptive immune system acting as the second line of defense [1]. The primary defense mechanisms of macrophages against pathogen infection are phagocytosis and cytokine production; pathogens and antigens can induce an inflammatory response [2]. Lipopolysaccharides (LPS), an endotoxin antigen from gram-negative bacteria, elicit a flaring inflammatory response from macrophages [2], which are the primary target cells infected by the obligate intracellular gram-negative bacterium *Orientia* (*O*.) *tsutsugamushi* in human skin [3, 4]. Scrub typhus, caused by *O*. *tsutsugamushi*, is transmitted to humans via the bite of infected mites, often resulting in severe complications, including adult respiratory distress syndrome (ARDS), acute renal failure, acute hepatic failure, and multiple organ dysfunction syndrome (MODS) [5–7]. The pathogenesis of ARDS and MODS is induced by a cytokine storm, a strong inflammatory response that spirals out of control [8, 9]. Consequently, a major goal of infection therapy is to provide a mechanism to regulate cytokine production and to determine the course of pathogenesis in clinical infections.

It has been established that Ca^2+^ signaling regulates the production of a variety of cytokines as part of a severe inflammatory response that results from complicated pathogen infections [10]. Although clinical studies have led to the development of new therapeutic approaches against severe inflammation and sepsis, the major focus has been on decreasing the cytokine storm, especially tumor necrosis factor (TNF)-α production [11–13]. Blocking TNF-α production is thought to be crucial for improving cell survival in cecal ligation puncture sepsis and intra‐abdominal sepsis [12, 13], because TNF-α facilitates the induction of cell injury via the activation of caspase/p38 and JNK MAP kinase cascades [14]. Ca^2+^ elevation, especially due to store-operated Ca^2+^ entry (SOCE), is also involved in TNF-α release from microglial cells under chronic purinergic stimulation [15]; however, the mechanism of Ca^2+^ signaling in regulating TNF-α production under pathogen infection has not been clearly demonstrated.

The present study hypothesizes that Ca^2+^ signaling is essential in regulating TNF-α production under *O*. *tsutsugamushi* infection. The proposed mechanism is that Ca^2+^ signaling disrupts intracellular Ca^2+^ elevation following a decrease of TNF-α production in macrophages. Although SOCE inhibitors such as 2-aminoethoxydiphenyl borate (2-APB) and SKF96365 have been found to block the activation of the mitogen-activated protein kinase (MAPK) pathway and Ca^2+^ signaling in neutrophils [16], the effect of 2-APB was not the same as that shown in macrophages. Our results indicate that 2-APB not only decreases SOCE activity to regulate TNF-α production, but also upregulates heat shock protein 70 (HSP70) to reduce TNF-α expression via the activation of the MAPK/ERK pathway in *O*. *tsutsugamushi*-infected macrophages. We propose in this report that there is a novel pathway for 2-APB to regulate pathogen-induced TNF-α production in macrophages and that an inhibitory mechanism against pathogen infection by 2-APB mitigates a cytokine storm during a severe inflammatory response.

## Materials and Methods

### Infection of macrophages by *O*. *tsutsugamushi*

Human monocytic THP-1 cells were purchased from the Taiwan Bioresource Collection and Research Center (BRCR, Taiwan). RPMI 1640 medium, which contained 10% (vol/vol) fetal bovine serum (FBS), 2 mM L-glutamine, 10 mM HEPES, 25 mM glucose, 1% (vol/vol) penicillin-streptomycin, and 1 mM sodium pyruvate, was prepared to maintain the THP-1 monocytes in a humidified 5% CO_2_ and 37°C incubator. 100 ng/mL phorbol 12-myristate 13-acetate (PMA, Sigma Aldrich) was utilized to induce THP-1 monocyte differentiation into macrophages. 2×10^5^ cells were seeded into a 6-well plate with 100 ng/mL PMA. After 48 h, the cells were washed three times with PBS and cultured overnight in fresh RPMI 1640 medium containing 2% FBS. The PMA-induced macrophages were infected with *O*. *tsutsugamushi*, TW-1 strain, which belongs to Karp strains, the most common (33.6%) and a highly virulent strain in Taiwan [17]. This strain was received from the Taiwan Centers for Disease Control. The methods used for TW-1 culture, isolation, and quantification can be found in our previous study [18]. THP-1-induced macrophages were infected with a high infection dose (one macrophage infected by 100 pathogens) [18].

### Tracing of *O*. *tsutsugamushi*

The effective invasion of *O*. *tsutsugamushi* into macrophages was determined by labelling macrophages with CellTracker^®^ Green Fluorescent Probe (Lonza) and labeling *O*. *tsutsugamushi* with CellTracker^™^ Red CMTPX (Invitrogen) in living status, respectively, before infection. The infected cells were fixed with 4% paraformaldehyde at 0, 15, 30, 45, and 60 min and nuclei were stained with DAPI (Sigma-Aldrich). The dynamics of intracellular infection were detected using an Olympus FV1000 confocal microscope equipped with an UPLanApo 100× objective lens [18].

### Calcium imaging

Ca^2+^ mobility was estimated by application of thapsigargin (TG; Sigma-Aldrich), according to methods previously described [19]. Before the experiments, cells were stained with 1 μM Fluo-4-AM (Molecular Probes) at 37°C for 20 min and then washed with balanced salt solution (BSS) buffer (5.4 mM KCSl, 5.5 mM d-glucose, 1 mM MgSO_4_, 130 mM NaCl, 20 mM Hepes pH 7.4, and 2 mM CaCl_2_). Intracellular Ca^2+^ concentrations were determined based on the ratio of fluorescence intensities. The intracellular Ca^2+^ concentration was calculated using calibration curves as previously described [19].

### Quantitative reverse transcription polymerase chain reaction (qRT-PCR)

Total RNA was extracted from pathogen-stimulated macrophages with Trizol reagent (Invitrogen). Reverse transcriptase reactions required 1 μg of RNA to synthesize complementary cDNA using an RT kit (Invitrogen). Incubation conditions were 10 min at 25°C, 120 min at 37°C, and 5 min at 85°C. The resulting cDNAs were used to identify the TNF-α expression level with the quantitative polymerase chain reaction (PCR) utilizing the SybrGreen PCR Master Mix Kit (Applied Biosystems, Carlsbad, CA, USA) and specific primers: TNF-α forward: CCC AGG GAC CTC TCT CTA ATC A and reverse: GCT ACA GGC TTG TCA CTC GG; GAPDH (GenBank accession number, NM_ 002046), forward: TGC ACC ACC AAC TGC TTA GC and reverse: GGC ATG GAC TGT GGT CAT GAG. Thermal cycling was conducted in an Applied Biosystems 7900HT fast real-time PCR system using the following cycling conditions: 95°C for 10 min, and 40 cycles at 95°C for 5 s, and 60°C for 30 s. Each complete amplification stage was followed by a dissociation stage at 95°C for 15 s and 60°C for 30 s [20].

### Cell viability assay

THP-1-induced macrophages were incubated with pathogens (*O*. *tsutsugamushi* or 1 μg/mL LPS) or co-incubated with 2-APB, PD98059, or SKF96365 for 24 h. Cell viability was determined by 3-(4,5-dimethyl-2-thiazolyl)-2, 5-diphenyl-2H-tetrazolium bromide (MTT) assay (Sigma-Aldrich) at a final concentration of 500 μg/mL in phosphate-buffered saline (PBS; Gibco), and incubation in the dark at 37°C for 3 h. After the resulting formazan crystals were dissolved by incubation with dimethyl sulfoxide at 37°C for 5 min, the solution was transferred to a 96-well ELISA plate and read at 570 nm in an ELISA reader.

### Western blot analysis

Total cell lysates (100 μg) were analyzed using SDS-PAGE on a 12% gel. After electro-blotting to a nitrocellulose membrane, membranes were blocked with 1% BSA for 1 h at room temperature. Membranes were washed with 0.1% TBST three times and then incubated with primary antibodies overnight at 4°C. Antibodies against Orai1(Merck Millipore), STIM1(OriGene), TRPV1 (Novus Biologicals), phospho-ERK (Cell Signaling Technology), ERK (Cell Signaling Technology), phosphor-JNK (BD Transduction Laboratories^™^), JNK (BD Transduction Laboratories^™^), phosphor-p38 (BD Transduction Laboratories^™^), p38 (BD Transduction Laboratories^™^), HSP10 (Enzo Life Sciences), HSP40 (Enzo Life Sciences), HSP70 (Enzo Life Sciences), HSP90 (Calbiochem, Merck Millipore), and β-actin (Santa Cruz) were utilized as the primary antibodies. The membranes were then treated with horseradish peroxidase-conjugated secondary antibodies (Amersham Biosciences). Immunoreactive proteins were visualized using enhanced chemiluminescence reagents (Amersham Biosciences).

### HSP70 knockdown

THP-1-induced macrophages were treated with 40 μM HSP70 siRNA (Santa Cruz) and GenMuteTM siRNA transfection reagent (SignaGen Laboratories) in accordance with the manufacturer’s protocol for 24 h, then incubated with pathogens (*O*. *tsutsugamushi* or LPS) or co-incubated with 2-APB for 24 h. Knockdown HSP70 in pathogen-stimulated macrophage assays was performed to further identify TNF-α production.

### Immunofluorescence assay

The ratio of the NF-κB translocation was determined with an immunofluorescence assay using an antibody against NF-κB (Santa Cruz). Pathogen-stimulated THP-1 was cultured on 24-mm coverslips in 35-mm 6-well plates. After three washes with PBS, the cells were fixed by incubation with 4% paraformaldehyde for 10 min. The fixed cells were then briefly washed with PBS and incubated overnight at 4°C in PBS containing 5% goat serum and 1% BSA with the appropriately diluted monoclonal antibody, NF-κB. After three washes with PBS, the cells were incubated for 1 h at room temperature with Alexa-488-conjugated goat anti-mouse IgG (Invitrogen) for NF-κB. The coverslips were washed three times with PBS (5 min each) and counterstained with 500 ng/mL 4,6-diamidino-2-phenylindole (DAPI, Sigma Aldrich) for 3 min. The coverslips were slide-mounted with an antifade mounting solution and imaged using an Olympus FV1000 laser scanning microscope [20].

### Statistical analysis

GraphPad Prism (La Jolla, CA) was used to generate bar charts, where error bars indicate standard deviations. One-way, two-tailed analysis of variance (ANOVA) was utilized to compare the means of each group. A *p*-value of less than 0.05 for differences between groups was considered statistically significant.

## Results

### 2-APB but not SKF96365 inhibited *O*. *tsutsugamushi*-induced intracellular Ca^2+^ elevation

**Fig 1:**
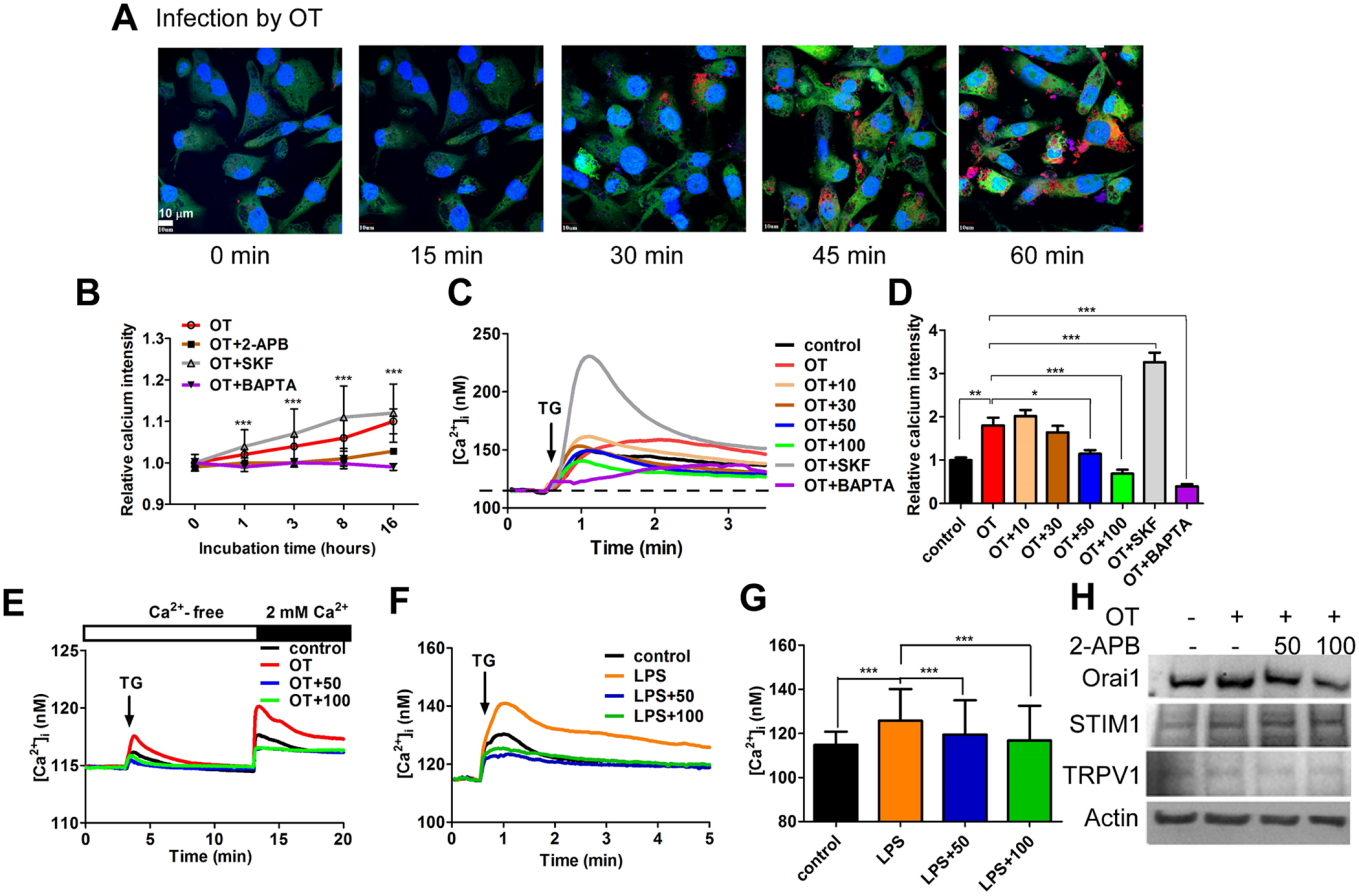
2-aminoethoxydipheny (2-APB) reduced pathogen-activated Ca^2+^ signaling in macrophages. (A) Fluorescence images of *O*. *tsutsugamushi* (OT)-infected macrophages at indicated time point with staining by Celltrackers (cytoplasm, green; OT, red) and DAPI (uncles, blue). (B) In *O*. *tsutsugamushi*-infected macrophages co-treatment with various Ca^2+^ inhibitors, 50 μM 2-APB (OT+2-APB), 50 μM SKF96365 (OT+SKF), or 25 μM BAPTA (OT+BAPTA) at indicated time points. (C) Effect of Ca^2+^ inhibitors, 2-APB (10 μM, 30 μM, 50 μM and 100 μM), SKF96365 (SKF) and BAPTA on intracellular Ca^2+^ concentration of *O*. *tsutsugamushi*-infected macrophages after 24 h of co-incubation. OT+10, OT+30, OT+50 and OT+100 indicate *O*. *tsutsugamushi* co-incubation with different concentration of 2-APB. Ca^2+^ imaging analysis of TG-induced Ca^2+^ response after application of 1 μM TG (small black arrow) (n = 3). (D) Quantification of area (above dotted line) of intracellular Ca^2+^ responses shown in (C) (*, *p* < 0.05; **, *p* < 0.01; ***, *p* < 0.001). (E) CaCl_2_ was extracellularly applied (large black bar) to enter Ca^2+^ via store-operated calcium channel after application of TG (small black bars) in Ca^2+^-free BSS solution (open bar) (n = 3). Effect of 2-APB on (F) Ca^2+^dynamics and (G) intracellular Ca^2+^ elevation in LPS-stimulated macrophages during 24 h of co-incubation (***, *p* < 0.001). LPS co-incubated with 50 μM 2-APB (LPS+50) and 100 μM 2-APB (LPS+100) respectively. (H) Orai1 expression was decreased by 2-APB, but not STIM1, TRPV1, or Actin in *O*. *tsutsugamushi*-infected macrophages, as determined by western blot analysis.

SOCE is crucially involved in modulating the pathogenesis of inflammation in murine peritoneal macrophages; blocking Ca^2+^ influx with SKF96365 impaired LPS- and IFN-γ-induced cytokine production [21]. To clarify whether SOCE is important for regulating cytokine production in the infection process by *O*. *tsutsugamushi*, we first compared the effect of SOCE inhibitors 2-APB and SKF96365 on Ca^2+^ influx in pathogen-infected macrophages. Cell-Tracker-Red-labeled *O*. *tsutsugamushi* at a dose of 100 pathogens per cell (high dose) [18] were used to infect human THP-1-induced macrophages. The time course of the infection process was then traced using a confocal microscope. Fluorescence images of macrophages with *O*. *tsutsugamushi* taken after 1 h of incubation are shown in Fig 1A. Slightly increased intracellular Ca^2+^ elevation by infection was suppressed with 2-APB, as indicated in Fig 1B. After 24 h of incubation with *O*. *tsutsugamushi*, a transient Ca^2+^ elevation induced by TG was found to be increased 1.7-fold, while applications of 50 and 100 μM 2-APB decreased the TG-induced Ca^2+^ response (Fig 1C and 1D). 2-APB also blocked *O*. *tsutsugamushi*-induced intracellular Ca^2+^ elevation at the indicated time point (Fig 1B); this suppression was mainly due to the mitigation of SOCE activated by *O*. *tsutsugamushi* (Fig 1E). Similar results are shown in Fig 1F and 1G, where the induction of intracellular Ca^2+^ elevation by LPS treatment for 24 h was significantly reduced with 2-APB. Unexpectedly, SKF96365, which has been reported to inhibit LPS- and LPS-plus-IFN-γ-induced Ca^2+^ influx [21], promoted *O*. *tsutsugamushi*-induced intracellular Ca^2+^ elevation (Fig 1B, 1C and 1D). According to a previous report, overexpression of Orai1 and STIM1 proteins influences the function of SOCE [22], and thus we further examined whether 2-APB affected Orai1 and STIM1 expressions under *O*. *tsutsugamushi* infection. Fig 1H indicates that pathogenic infection caused the induction of upregulated expression of Orail and STIM1, yet only Orai1 was attenuated to some extent by 2-APB. No effect was found for other Ca^2+^ entry channels, such as TRPV1 expression, which is known to be activated by 2-APB stimulation [23]. It is reasonable to conclude that 2-APB interrupted the pathogen-induced increase of intracellular Ca^2+^ concentration via the inhibition of SOCE activity in macrophages.

### 2-APB inhibited pathogen-induced TNF-α production

**Fig 2:**
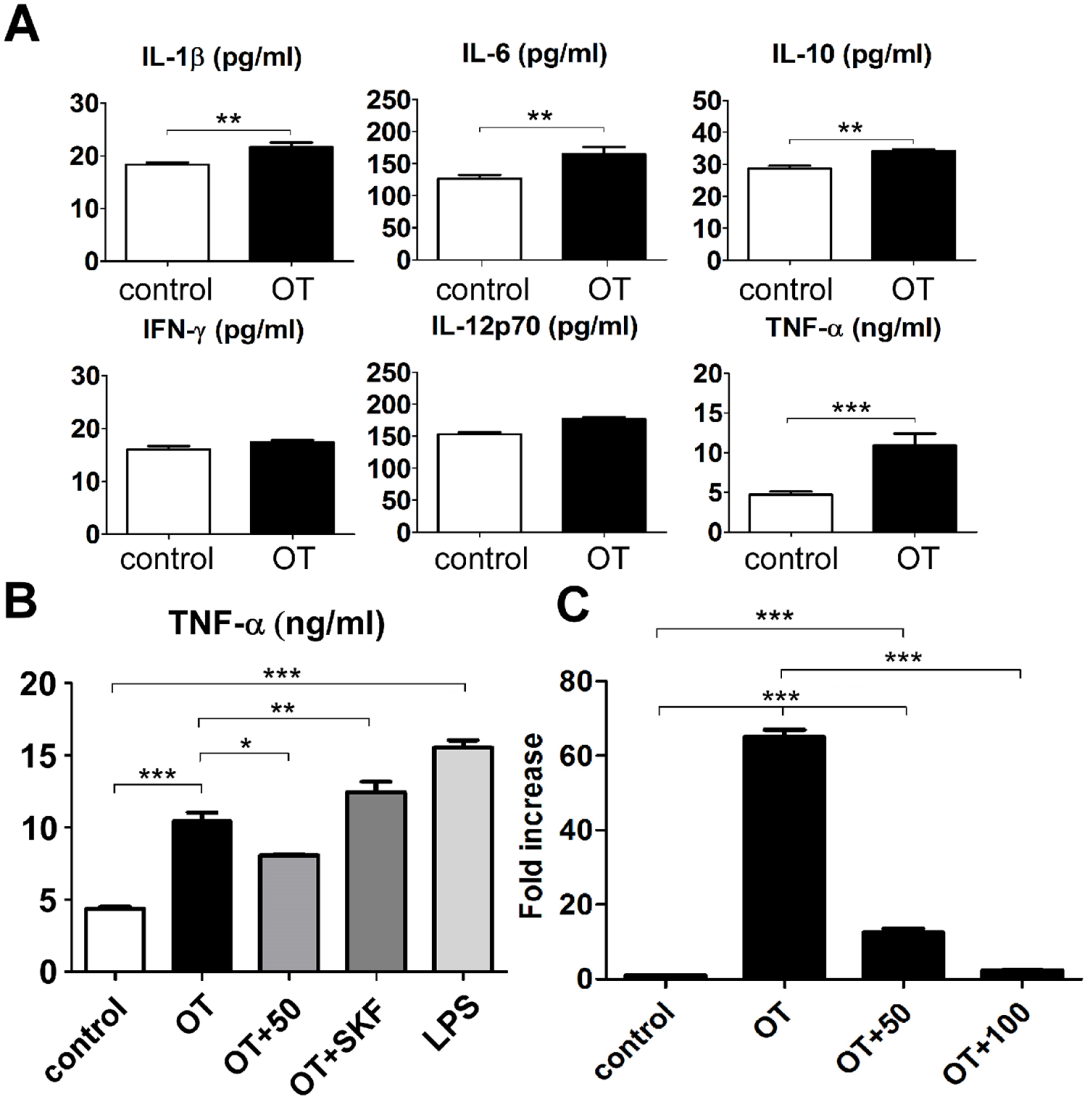
Effects of *O*. *tsutsugamushi* on cytokine production by macrophages. (A) Production of various cytokines was measured with ELISA system (eBioscience) at 24 h after infection (**, *p* < 0.01; ***, *p* < 0.001). 2-APB (50, 50 μM and 100, 100 μM) reduction of *O*. *tsutsugamushi*-induced increase of TNF-α expression was analyzed by (B) ELISA and (C) qRT-PCR in macrophages (*, *p* < 0.05; **, *p* < 0.01; ***, *p* < 0.001).

Next, we determined the pathogen-induced production profiles of inflammatory cytokines, namely IL-1β, IL-6, IL-10, INF-γ, IL-12p70, and TNF-α, with *O*. *tsutsugamushi* infection for 24 h. The production of IL-1β, IL-6, IL-10, and TNF-α increased, especially that of TNF-α, which dramatically increased 2-fold relative to the control (Fig 2A). We then confirmed whether pathogen-activated TNF-α expression was repressed by SOCE inhibitors 2-APB and SKF96365. Similar to the results for TG-induced Ca^2+^ elevation shown in Fig 1C and 1D, SKF96365 was unable to decrease *O*. *tsutsugamushi*-induced TNF-α production and even promoted it, even though its concentration was sufficiently high (50 μM) (Fig 2B and 2C). Previous reports indicated that SOCE inhibitors SKF96365 and 2-APB downregulated TNF-α release under conditions of chronic stress [15], while our results demonstrate that 2-APB reduced not only TNF-α release, but also TNF-α expression (Fig 2B and 2C). The TNF-α promoter has DNA binding elements with Ca^2+^-dependent transcription factors [25], such as NF-κB [26] or cAMP response element-binding protein (CREB) [27]. When intracellular Ca^2+^ elevation occurs, these transcription factors will translocate to the nucleus.

**Fig 3:**
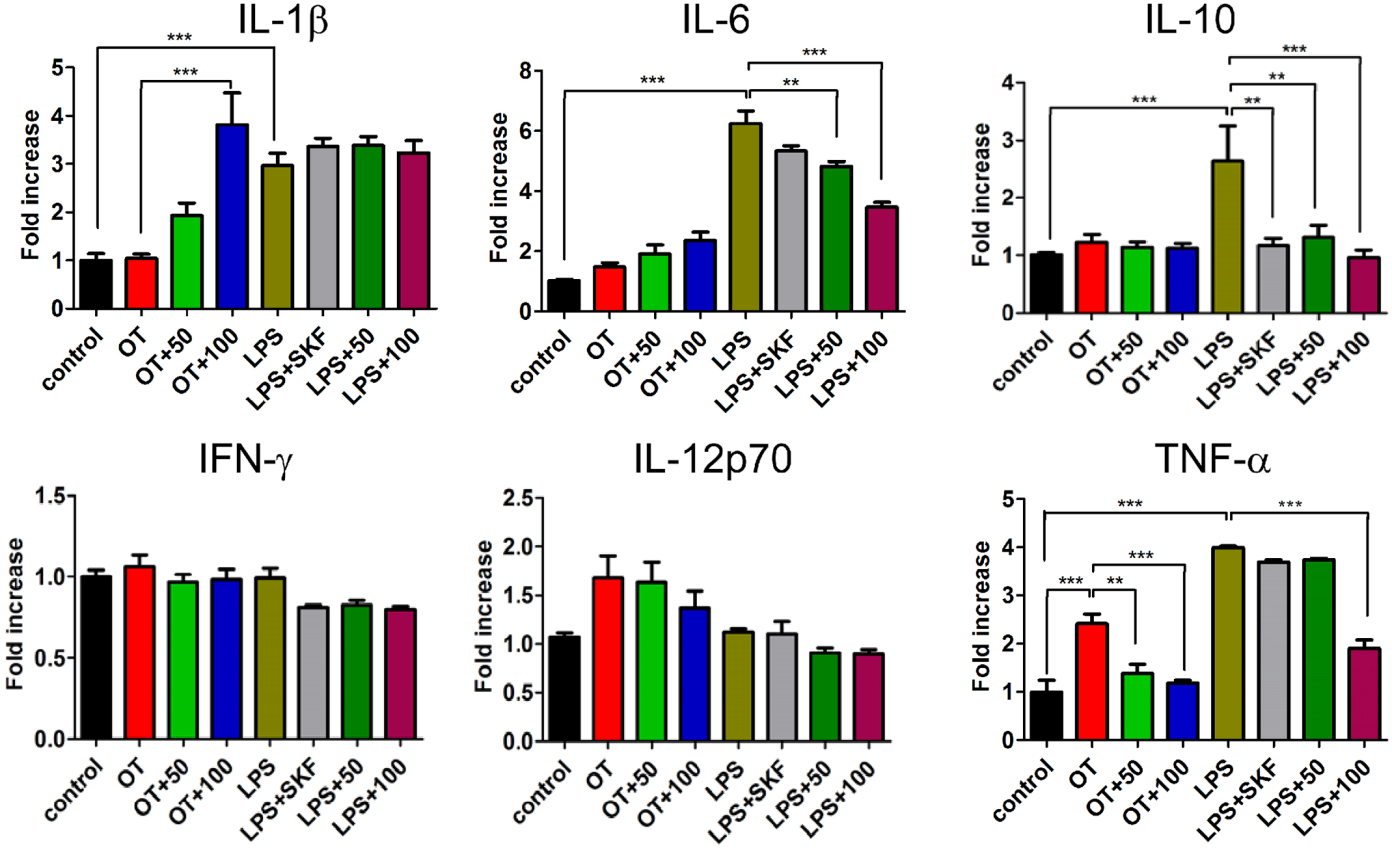
Effect of 2-APB on profiles of inflammatory cytokines in *O*. *tsutsugamushi*-infected and LPS-activated macrophages. Cytokine levels of production were determined with ELISA system (eBioscience) analysis during 24 h of co-treatment (*, *p* < 0.05; **, *p* < 0.01; ***, *p* < 0.001).

Therefore, it was not unexpected that 2-APB mitigated TNF-α production and expression. Additionally, we further analyzed the profiles of inflammatory cytokine production by stimulating macrophages with *O*. *tsutsugamushi* or LPS. Interestingly, in the case of infection by *O*. *tsutsugamushi*, 2-APB significantly activated IL-1β production (Fig 3), which is known to be important for the activation of adaptive immune cells, namely antigen-specific T cells [24]. 2-APB also slightly increased the amounts of IL-6 and IL-12p70, which are known to modulate Type 2 immune responses [25, 26]. Compared with the systemic innate immune response, the adaptive immune system specifically targets pathogens, and normalizes or eliminates pathogen activation [27]. The obligate intracellular bacterium *O*. *tsutsugamushi* targets and infects macrophages: IL-1β elevation by 2-APB provides an efficient strategy to mitigate this infection. More significantly, 2-APB was found to downregulate the induction of TNF-α production through stimulated by LPS or *O*. *tsutsugamushi* (Fig 3).

### ERK pathway involved in 2-APB-induced increase in level of HSP70 expression in *O*. *tsutsugamushi-*infected macrophages

**Fig 4:**
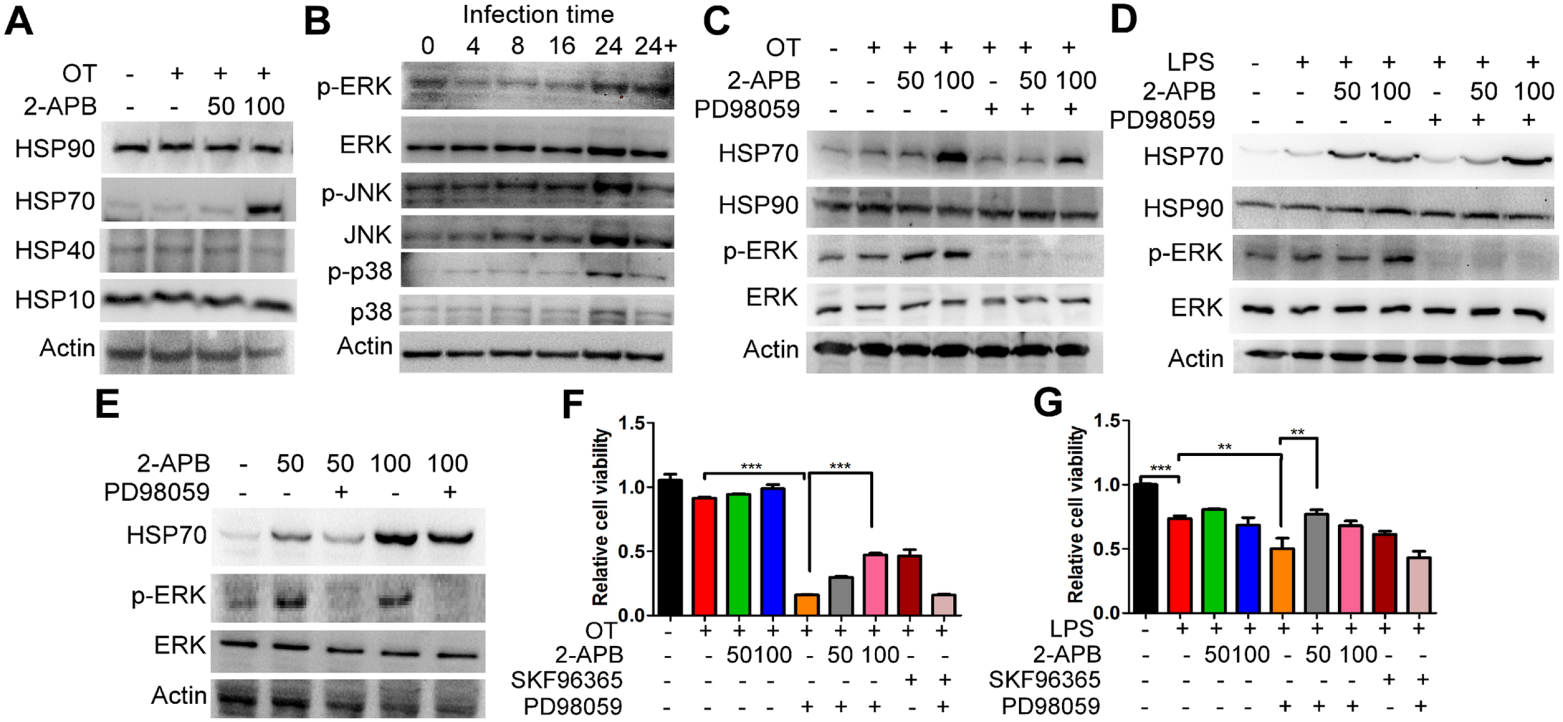
Increased level of HSP70 expression by treatment with 2-APB in *O*. *tsutsugamushi*-infected or LPS-stimulated macrophages. (A) Effect of 2-APB on expressions of HSP10, HSP40, HSP70, HSP90, and Actin in *O*. *tsutsugamushi* (OT)-infected macrophages. (B) Western blot analysis was used to analyze MAPK signal pathways in *O*. *tsutsugamushi*-infected macrophages at indicated time point. Treatments included phosphorylated JNK (p-JNK), JNK, phosphorylated p38 (p-p38), and p38, which were blocked by co-treatment with 50 μM of 2-APB for 24 h (24+). Further measurements were made of expression of phosphorylated ERK (p-ERK), ERK, HSP70, and Actin, which were treated with MAP kinase (MEK) inhibitor, 50 μM PD98059, 2-APB (50, 50 μM and 100, 100 μM) or combination for 24 h in (C) *O*. *tsutsugamushi*-infected and (D) LPS-stimulated macrophages. (E) Western blot analysis of expression of ERK pathway, HSP70, and Actin without pathogen infection in macrophages. Survival probability is shown for each condition in panel with (F) *O*. *tsutsugamushi*-stimulated macrophages and (G) LPS-stimulated macrophages for 24 h (**, *p* < 0.01; ***, *p* < 0.001).

According to the results described above, 2-APB reduced the level of TNF-α production by compressing Ca^2+^ entry under pathogen infection in macrophages. Since induction of HSP70 has also been found to be involved in modulating TNF-α production [28], inhibited TNF-α production was examined to determine a potential relationship to bacterial LPS stimulation in monocytes or macrophages [29]. Interestingly, only HSP70 was upregulated by 2-APB when we examined whether 2-APB affected expressions of HSP10, HSP40, HSP70, and HSP90 under *O*. *tsutsugamushi* infection (Fig 4A). The results imply that several signal transduction pathways are activated by 2-APB to promote HSP70 expression. Therefore, we further investigated the signaling pathway for 2-APB-induced upregulation of HSP70. We incubated macrophages with *O*. *tsutsugamushi* or co-incubated them with *O*. *tsutsugamushi* and 2-APB, and then analyzed MAPK signal pathways at the indicated time points. As shown in Fig 4B, expressions of JNK and p38 were attenuated by 2-APB but that of ERK was unexpectedly increased. Simultaneously, interruption of the enhancements of ERK and HSP70 was revealed using the MAPK/ERK inhibitor PD98059 (Fig 4C). Unlike with LPS treatment, PD98059 did not interrupt HSP70 and even promoted its expression with 100 μM 2-APB, despite disruption of HSP70 production by PD98059 and 50 μM 2-APB co-treatment (Fig 4D). 2-APB-induced upregulation of HSP70 was found to be dependent on ERK pathway activity. Without pathogen infection, PD98059 attenuated the increased-HSP70 by 50 μM or 100 μM 2-APB applications (Fig 4E). Interestingly, these increases of HSP70 did not increase cell viability under pathogenesis (Fig 4F and 4G). Unexpectedly, 2-APB also promoted the ERK pathway in macrophages, and cell viability was extended after by PD98059 (Fig 4F and 4G). Thus, 2-APB-induced upregulation of HSP70 cannot enhance cell survival but it could be crucial to the activation of the ERK pathway regulation of HSP70 expression in *O*. *tsutsugamushi*-infected macrophages.

### 2-APB-induced upregulation of HSP70 against *O*. *tsutsugamushi*-activated TNF-α expression

**Fig 5:**
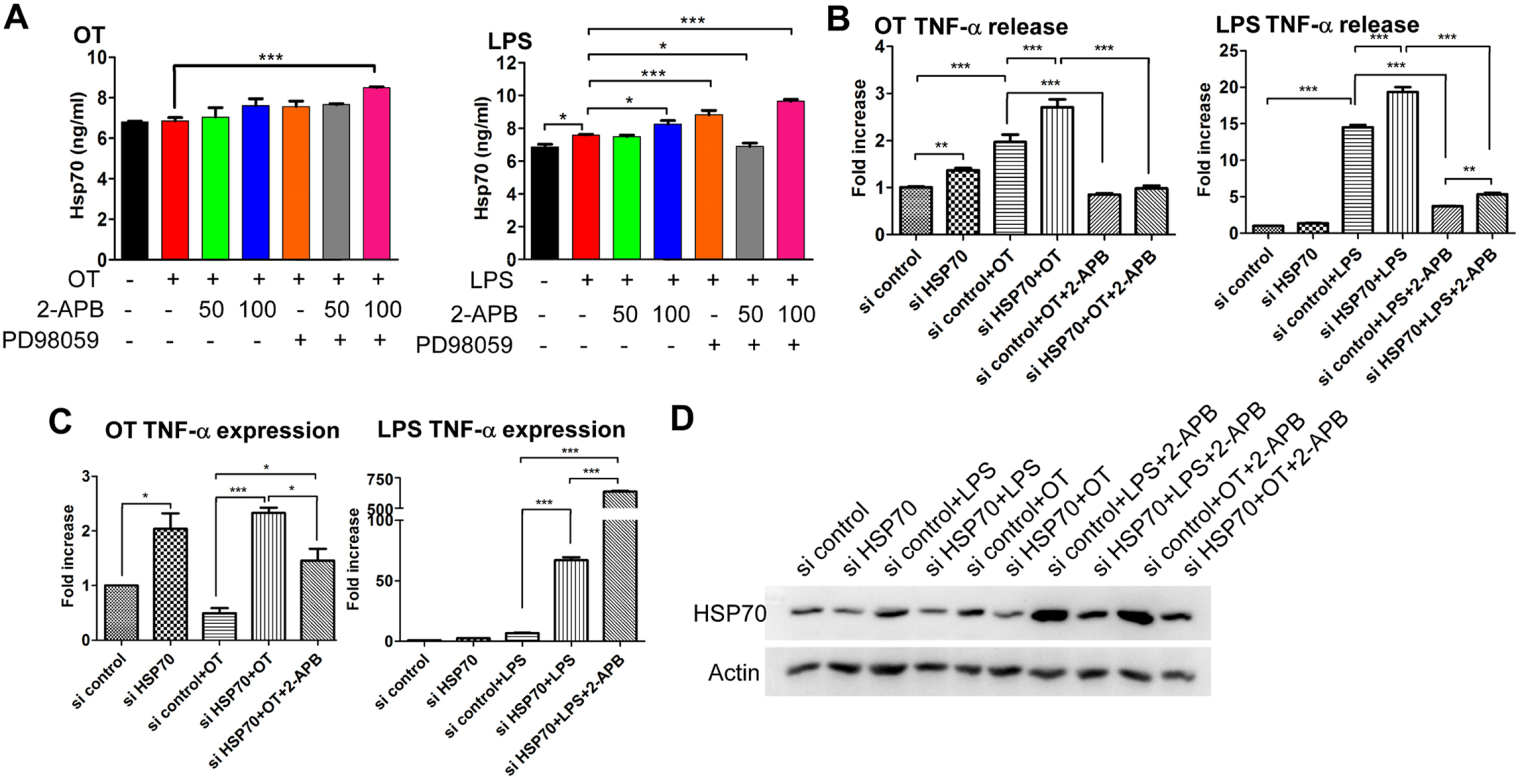
*O*. *tsutsugamushi*-activated TNF-α production was interrupted by increased level of HSP70 in macrophages. (A) Cells were co-incubated with pathogens and 2-APB or PD98059 for 24 h. There was no effect on HSP70 production after treatment with 2-APB or PD98059 in *O*. *tsutsugamushi*-infected and LPS-activated macrophages, as determined using ELISA system (Enzo Life Sciences) (*, *p* < 0.05; ***, *p* < 0.001). HSP70 was knocked down by HSP70 siRNA, and then co-treated with pathogens and 2-APB for 24 h. (B) TNF-α release and (C) TNF-α expression were measured using ELISA system and qRT-PCR analysis, respectively (*, *p* < 0.05; **, *p* < 0.01; ***, *p* < 0.001). (D) Western blot analysis of expression of HSP70 shown in (B) and (C).

The majority of data reported so far indicates that HSP70 is a protective system against endotoxin-induced cell damage [30]. This defense mechanism is used along with the disruption of the NF-κB/TNF-α axis [28, 29]. We did a follow-up to demonstrate whether 2-APB increased HSP70-influenced TNF-α expression. However, because exogenous HSP70 also has a therapeutic effect against endotoxin manifestations [31], we initially measured HSP70 concentration under various conditions of media. As shown in Fig 5A, there was no difference in HSP70 production under the various conditioning treatments. This figure also demonstrates that activated pathogens or 2-APB-induced HSP70 were not secreted to the outside. To find out how HSP70 affects TNF-α production, we performed a knockdown of HSP70 and further identified the release and expression of TNF-α. Consistent with the results for *O*. *tsutsugamushi*-infected macrophages shown in Fig 3, blocked HSP70 expression along with *O*. *tsutsugamushi* infection stimulated TNF-α production, whose dramatically increased level was attenuated by 2-APB treatment (Fig 5B and 5C). However, there was a difference between the release and expression of TNF-α with LPS stimulation; although 2-APB eliminated TNF-α release, TNF-α expression levels were not reduced, and even increased, after knockdown of HSP70 (Fig 5B and 5C). Increases in the level of HSP70 by 2-APB were found in knockdown HSP70 (Fig 5D). An unclear mechanism for 2-APB inducible HSP70 increased TNF-α expression with endogenous HSP70 knockdown in LPS-stimulated macrophages, but not all of the upregulated TNF-α was released to the outside due to 2-APB mitigating SOCE. Interestingly, with pathogen stimulation, the activation of NF-κB in knockdown HSP70 was higher than that in the control, but restore HSP70 by 2-APB reduced the ratio of NF-κB translocation (S1 Fig). It could be that NF-κB drives signaling pathway is not major in LPS-induced TNF-α expression. 2-APB may synergistically activate several signaling pathways to promote TNF-α expression with LPS. As a result, 2-APB strategically mediates pathogen-activated TNF-α production in a way that is decreasing the release by pitting SOCE and upregulating HSP70 to repress NF-κB activity against TNF-α expression in *O*. *tsutsugamushi*-infected macrophages.

## Discussion

There is an established protocol for a pathogen-induced severe inflammatory response. The primary response is treatment with antibiotics and stabilization of the patient’s condition in the event of a strong cytokine storm. A new therapeutic approach has recently been proposed, one that helps the body inhibiting TNF-α production by each inhibitor or anti-TNF-α antibody for patients recovering from serious inflammation [11]. Many studies have also supported that Ca^2+^ entry, especially via SOCE, is integrally involved in an inflammatory response or inflammation-induced TNF-α production [32]. Treatments that help prevent elevation of intracellular Ca^2+^ may be efficient for severe inflammation. 2-APB is a popular SOCE inhibitor that has been used in various Ca^2+^ signaling studies. However, except for Ca^2+^ signaling via SOCE inhibitors, there is little information on the possible mechanism of 2-APB against an inflammatory response, although Ca^2+^ signaling is the underlying mechanism for regulating the inflammatory process. The present study found a new mechanism of 2-APB for inhibiting inflammation-induced TNF-α production via upregulation of the ERK/HSP70 axis in *O*. *tsutsugamushi*-infected macrophages. In addition, 2-APB also elicited HSP70 overexpression in LPS-stimulated macrophages, but the importance for 2-APB governed LPS-induced TNF-α production is to repress TNF-α release outside of the cells. Based on our results, despite having similar pathogenesis patterns of inflammation, *O*. *tsutsugamushi* and LPS have distinct differences in their pathogenic mechanisms. In *O*. *tsutsugamushi* infection, 2-APB suppressed expression of TNF-α, whereas in LPS infection, 2-APB increased the TNF-α level (Fig 5C); yet, for both pathogens, TNF-α was not released to the outside due to 2-APB mitigation of SOCE (Fig 5B). Interestingly, TNF-α is secreted initially in a membrane-bound form (vesicle), and then is transformed into a water soluble form by TNF-α converting enzyme (TACE) [33]. It is possible that TNF-α is bound on the vesicle membrane because of an insufficient level of intracellular Ca^2+^ due to the inhibition of SOCE by 2-APB. So far, there is no direct evidence of the importance of Ca^2+^ concentration in regulating TACE; this hypothesis will be tested in the near future.

HSP70 was induced via activation of the ERK pathway [34] and 2-APB-stimulated ERK activity [35] has been previously reported. Activated SOCE is also associated with activation of ERK signaling and is inhibited by calmodulin kinase II (CaMKII) and Raf-1 in malignant melanoma [36]. In the present experiments, 2-APB treatment blocked SOCE but activated the EKR pathway to overexpress HSP70 in macrophages. Nevertheless, another signal transduction pathway joins the regulation of HSP70, because PD98059 cannot repress HSP70 completely. The Stat3/HSP70 axis [37] and the HIF/HSP70 axis [38] are candidate pathways involved in the 2-APB regulation of HSP70 upregulation. We found that 50 μM 2-APB treatment was followed by an increase of Stat3 expression in LPS-stimulated macrophages (data not shown). In contrast, 2-APB is known to suppress reactive oxygen species production, which results from inflammation-increased mitochondrial Ca^2+^ overloading [39]; consequently, the HIF/HSP70 axis may not be involved in 2-APB-mediated HSP70 overexpression. Interestingly, in Fig 4D, PD98059 inhibited 50 μM 2-APB-induced HSP70, but not 100 μM 2-APB-induced HSP70. Even though 2-APB facilitates resistance to TNF-α production under LPS stimulation, 50 and 100 μM 2-APB may use different pathways in LPS-induced macrophages.

Our experiments focused on 2-APB-induced overexpression of HSP70, which was expected to facilitate cell survival of macrophages. As shown in Fig 5A, however, HSP70 was not secreted to the outside to maintain an ongoing therapeutic effect. Furthermore, upregulated HSP70 was not expressed on the cell surface (data not shown), and thus it was impossible to protect granzyme B from NK cells [40]. An explanation of these conflicting data is that Ca^2+^ is the crucial factor that regulates the performance of HSP70, because HSP70 binds two Ca^2+^ ions within the ATPase domain to perform its chaperone function [41]. It is well known that 2-APB can reduce Ca^2+^ mobilization by blocking SOCE [42] and is able to block transient receptor potential canonical (TRPC) channels [20]. It is reasonable to consider that the disruption of Ca^2+^ mobilization by 2-APB negates the chaperone function of HSP7; however, in this case, the involvement of TRPC channels can be excluded because general TRPC channel inhibitor SKF96365 cannot block Ca^2+^ entry efficiently. Thus, SOCE may play a significant role in increasing intracellular Ca^2+^ concentrations through *O*. *tsutsugamushi* infection, as shown in Fig 1H (a schematic diagram of the increased level of Orai1 caused by *O*. *tsutsugamushi* infection). Similar results are also reported in neuron cells, LPS increases SOCE activation by enhancing the level of Orai1 [43].

Inflammation from injury, such as spinal cord injury or brain injury, promotes TNF-α production, altering macrophage populations of the M1/M2 phenotype [44]. Under TNF-α stimulation, the majority of M1 phenotype macrophages enhance the inflammatory response, but inflammation caused by protective M2 macrophages is eliminated because of the disruption in TNF-α production [44]. It is unknown whether 2-APB can switch the population of M1/ M2 macrophages during an inflammatory response. According to our study, 2-APB may affect the population of M1/M2 by blocking TNF-α production. As a result, once 2-APB increases the protective M2 macrophages, it may protective in the recovery of cell damage and maintain cell survival during severe inflammation.

*O*. *tsutsugamushi* is potentially lethal due to its induction of a serious inflammatory response and a cytokine storm. It also induces strong Type 1 cytokines (TNF-α, IFN-γ, and CXCL9-11), but impairs Type 2 cytokines (IL-7, IL-4, and IL-13) [45]. This is all implicated by the absence of specific antibodies against *O*. *tsutsugamushi* infection. 2-APB significantly downregulated TNF-α production, while slightly increased IL-6 and decreased IL-12p70 to mediate Type 2 immune responses (Fig 3) [25, 26]. In the face of poor patient outcome with treatment by clinical antibiotics, 2-APB or similar compounds can be developed as an efficient and therapeutic approach. Consequently, our further work will apply 2-APB as an *in vivo* treatment and investigate the effect of 2-APB on a pathogen-infected murine model.

**Fig 6:**
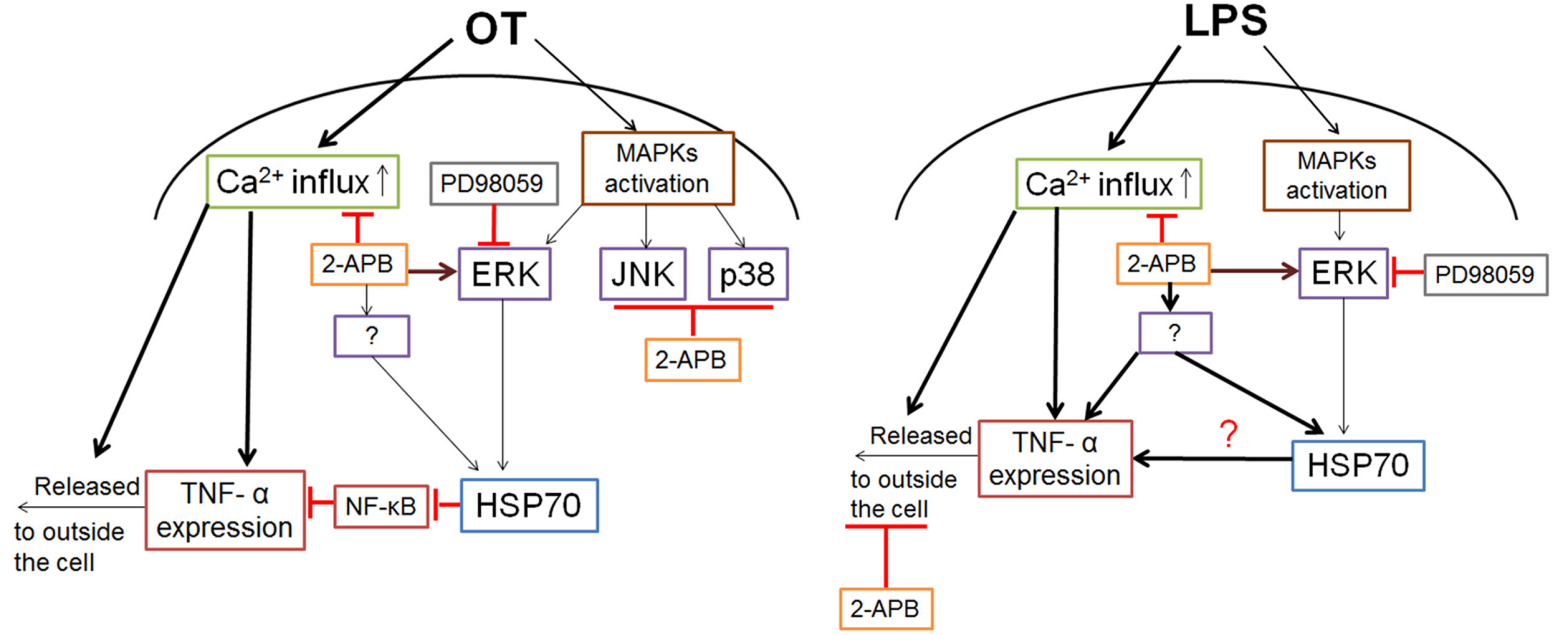
Schematic diagram of 2-APB activity in strategically regulating *O*. *tsutsugamushi*-induced and LPS-stimulated TNF-α production in macrophages. (A) Pathogenic infection induced increased level of TNF-α, which was attenuated by 2-APB interrupting Ca^2+^ signaling activity; suppressed Ca^2+^ mobilization also suppressed release and expression of TNF-α. Mitogen-activated protein kinase (MAPK) pathway is involved in regulating TNF-α production under *O*. *tsutsugamushi* infection; however, 2-APB restrains signal pathway of JNK and p38 but activates ERK pathway to promote upregulation of HSP70, which facilitates downregulation of TNF-α expression by blocking NF-κB translocation to nucleus. (B) Although 2-APB attenuates LPS-stimulated TNF-α production, different mechanism displays in regulating TNF-α expression. The importance of 2-APB in LPS-stimulated macrophages is to block TNF-α release rather than inducing ERK pathway to activate upregulation of HSP70.

Conclusively, 2-APB strategically decreases TNF-α production by utilizing its unique mechanism in pathogen-infected macrophages. It does this by downregulating Ca^2+^ influx to interrupt the release and expression of TNF-α and upregulating HSP70 to suppress TNF-α expression via activation of ERK or other pathways (Fig 6). Additionally, by increasing the number of adaptive-immunity-associated cytokines, IL-1β, IL-6, and IL-12p70, 2-APB efficiently attenuates a severe inflammatory response. Our findings show the potential for immune regulatory treatment in clinical studies using 2-APB.

## Supporting Information

S1 Fig Effect of 2-APB on NF-κB activation in *O*. *tsutsugamushi*-infected and LPS-activated macrophages. (A) Knocked down HSP70 promotes translocation of NF-κB (green) to nucleus (blue) with pathogenic stimulation by immunofluorescence analysis. (B) Activation of NF-κB quantified by measurement of fluorescent intensity of NF-κB in nucleus area using an Olympus fluorescence microscope with an average fluorescence intensity of more than 1000 cells (*, *p* < 0.05; ***, *p* < 0.001).
(TIF)
