# Supplementary figures and images for: A Novel Strategy for TNF-Alpha Production by 2-APB Induced Downregulated SOCE and Upregulated HSP70 in O. tsutsugamushi-Infected Human Macrophages
Source: PLoS One. 2016 Jul 29;11(7):e0159299. doi: 10.1371/journal.pone.0159299 (PMC4966960; doi:10.1371/journal.pone.0159299)

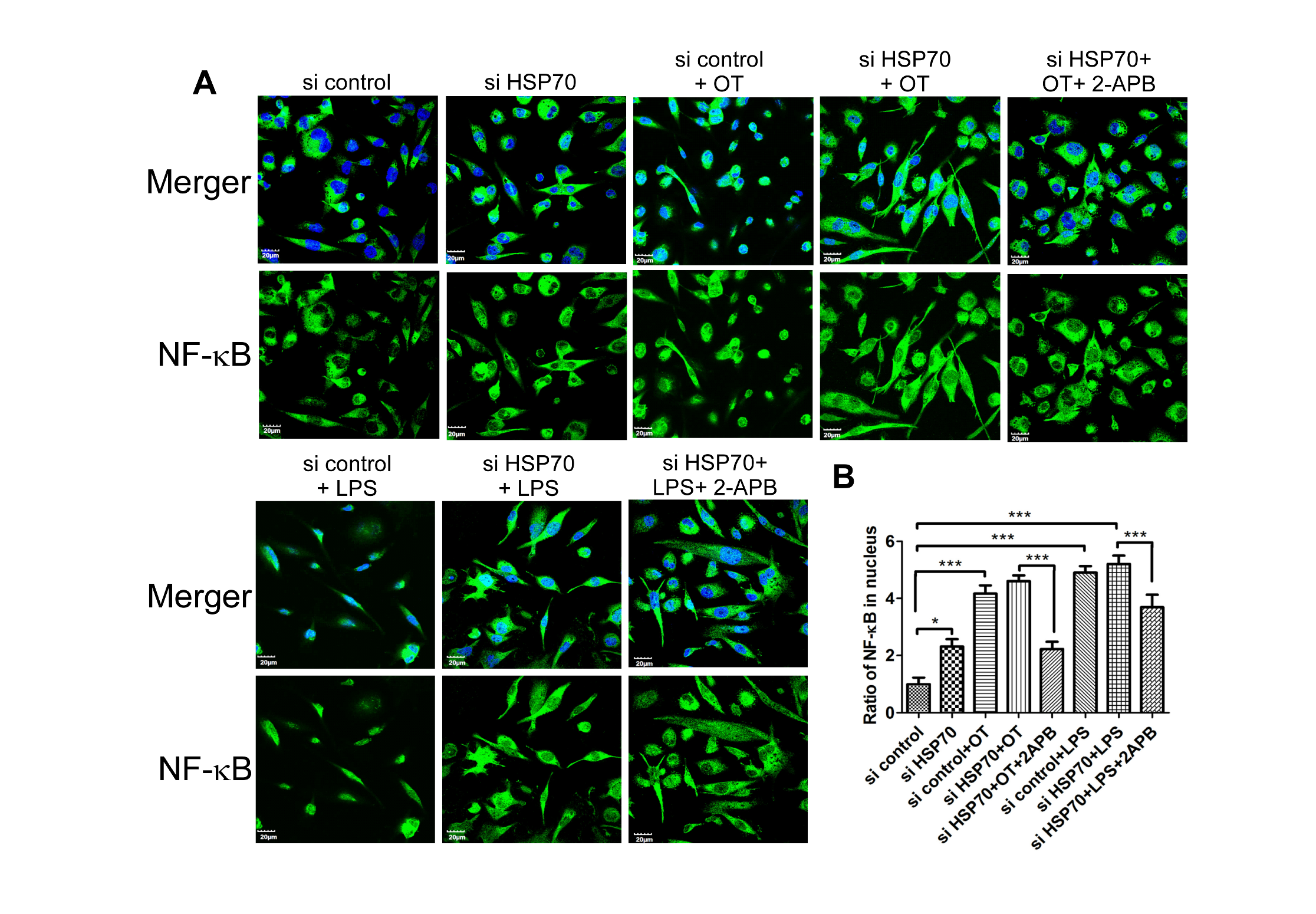

Supplement: S1 Fig — (A) Knocked down HSP70 promotes translocation of NF-κB (green) to nucleus (blue) with pathogenic stimulation by immunofluorescence analysis. (B) Activation of NF-κB quantified by measurement of fluorescent intensity of NF-κB in nucleus area using an Olympus fluorescence microscope with an average fluorescence intensity of more than 1000 cells (*, p < 0.05; ***, p < 0.001). (TIF) [file pone.0159299.s001.tif]
